# 5,5′-Bis[(2,2,2-trifluoro­eth­oxy)meth­yl]-2,2′-bipyridine

**DOI:** 10.1107/S1600536811000730

**Published:** 2011-01-12

**Authors:** Norman Lu, Wen-Han Tu, Wei-Hsuan Chang, Zong-Wei Wu, Han-Chang Su

**Affiliations:** a Institute of Organic and Polymeric Materials, National Taipei University of Technology, Taipei 106, Taiwan

## Abstract

The complete molecule of the title compound, C_16_H_14_F_6_N_2_O_2_, is generated by crystallographic inversion symmetry, which results in two short intramolecular C—H⋯N hydrogen-bond contacts per molecule. In the crystal, aromatic π–π stacking [centroid–centroid distance = 3.457 (2) Å] and weak C—H⋯π inter­actions occur. A short H⋯H [2.32 (3) Å] contact is present.

## Related literature

For related structures and background to the anti-planar geometry of bpy, see: Lu, Tu, Wu *et al.* (2010 ▶); Iyer *et al.* (2005 ▶); Heirtzler *et al.* (2002 ▶); Maury *et al.* (2001 ▶); Vogtle *et al.* (1990 ▶). For background to the bipyridine (bpy) ligand, see: Bain *et al.* (1989 ▶); Chambron & Sauvage (1986 ▶, 1987 ▶); Grätzel (2001 ▶); Haga *et al.* (2000 ▶); Lu, Tu, Hou *et al.* (2010 ▶); Lu, Tu, Wen *et al.* (2010 ▶); Lu *et al.* (2007 ▶). For C—H⋯H—C inter­actions, see: Wolstenholme & Cameron (2006 ▶).
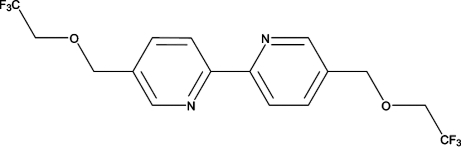


## Experimental

### 

#### Crystal data

C_16_H_14_F_6_N_2_O_2_
*M* *_r_* = 380.29
Triclinic, 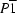

*a* = 4.6573 (2) Å
*b* = 5.6842 (3) Å
*c* = 15.7273 (8) Å
α = 94.298 (3)°
β = 98.473 (3)°
γ = 105.689 (4)°
*V* = 393.57 (3) Å^3^
*Z* = 1
Mo *K*α radiation
μ = 0.15 mm^−1^
*T* = 100 K
0.2 × 0.14 × 0.12 mm

#### Data collection

Bruker APEXII CCD area-detector diffractometer
Absorption correction: multi-scan (*SADABS*; Bruker, 2008 ▶) *T*_min_ = 0.664, *T*_max_ = 0.746
6627 measured reflections
1577 independent reflections
1348 reflections with *I* > 2σ(*I*)
*R*_int_ = 0.024

#### Refinement

*R*[*F* ^2^ > 2σ(*F* ^2^)] = 0.031
*wR*(*F* ^2^) = 0.104
*S* = 1.12
1577 reflections
146 parameters
All H-atom parameters refined
Δρ_max_ = 0.29 e Å^−3^
Δρ_min_ = −0.27 e Å^−3^

### 

Data collection: *APEX2* (Bruker, 2010 ▶); cell refinement: *SAINT* (Bruker, 2009 ▶); data reduction: *SAINT*; program(s) used to solve structure: *SHELXS97* (Sheldrick, 2008 ▶); program(s) used to refine structure: *SHELXL97* (Sheldrick, 2008 ▶); molecular graphics: *ORTEP-3 for Windows* (Farrugia, 1997 ▶); software used to prepare material for publication: *WinGX* (Farrugia, 1999 ▶).

## Supplementary Material

Crystal structure: contains datablocks I, global. DOI: 10.1107/S1600536811000730/fk2030sup1.cif

Structure factors: contains datablocks I. DOI: 10.1107/S1600536811000730/fk2030Isup2.hkl

Additional supplementary materials:  crystallographic information; 3D view; checkCIF report

## Figures and Tables

**Table 1:** Hydrogen-bond geometry (Å, °) *Cg* is the centroid of the N,C1–C5 ring.

*D*—H⋯*A*	*D*—H	H⋯*A*	*D*⋯*A*	*D*—H⋯*A*
C4—H4⋯N^i^	0.925 (17)	2.464 (18)	2.809 (2)	102.3 (12)
C6—H6*A*⋯*Cg*^ii^	0.990 (19)	2.59	3.5089 (16)	155

Symmetry codes: (i) 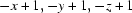; (ii) 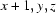.

**Fig. 1.**
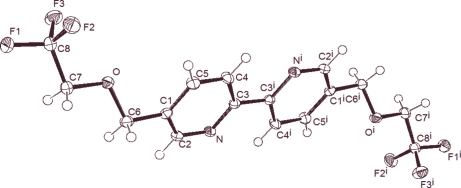
Molecular structure of I with displacement ellipsoids at the 50% probability level. [Symmetry code: (i) 1-x, 1-y, 1-z.]

**Fig. 2.**
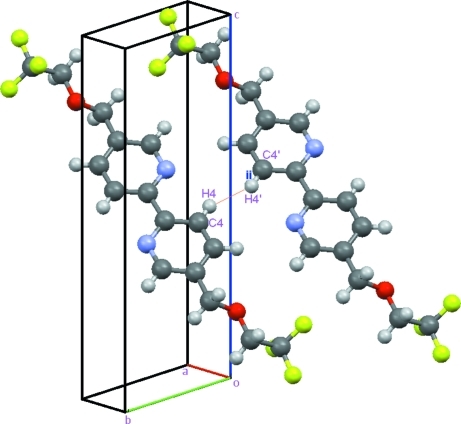
The rare C4–H4···H4'–C4' interaction on inversion related molecules; the H4···H4' distance and C4–H4···H4' angle are 2.32 (3) Å and 113 (2) °. [Symmetry code: (ii) 2-x, 1-y, 1-z.]

## supplementary crystallographic information

### Comment

Bipyridine (bpy) ligand is among the most versatile ligands in organometallics. It has been extensively used to prepare various chelating compounds with different metal ions (Haga *et al.*, 2000; Bain *et al.*, 1989; Grätzel, 2001; Chambron & Sauvage, 1987, 1986). Structures with the motif {[4,4'-bis(R_f_CH_2_OCH_2_) -2,2'-bpy]MCl_2_, M = Pd or Pt} are interesting and reveal the blue shifting C–H···F–C hydrogen bonding (Lu, Tu, Hou *et al.*, 2010; Lu, Tu, Wen *et al.*, 2010; Lu, *et al.*, 2007). However, the X-ray crystal structure of poly-fluorinated bpy ligands still remains elusive until recent elucidation of the structure on simplest 4,4'-bis(CF_3_CH_2_OCH_2_)-2,2'-bpy (Lu, Tu, Wu *et al.*, 2010). Reported here is the significantly different crystal structure on its 5,5'-isomer. They vary only on the positions of two identical substituents, yet features of packing in the solid state show little in similarity.

The title compound I is centro-symmetric and crystallizes in the space group of P-1, one half of the molecule being crystallographically independent. Two structural features of the title compound I are the planarity and the *anti* conformation of connected pyridyl units. The bpy exhibits a planar core and the N–C3–C3^i^–N^i^ torsion angle is 180°, similar to the values in its 4,4'-isomer (Lu, Tu, Wu *et al.*, 2010). Also noticed is the *intramolecular* weak hydrogen bonding interaction on C4–H4···N^i^, as suggested by the short H4···N^i^ distance of 2.46 (2) Å and the C4–H4···N^i^ angle of 102 (1)° (see Fig. 1).

Although both the title compound and its 4,4'-isomer (Lu, Tu, Wu *et al.*, 2010) have the same molecular formula and their bpy cores are similarly planar, their identical side chains, positioned differently, show very different *intermolecular* interactions and packing methods. The *intramolecular* C–H···O and *intermolecular* C–H···N and C–H···F interactions, which were observed in 4,4'-bis(2,2,2-trifluoroethoxymethyl)-2,2'-bipyridine (Lu, Tu, Wu *et al.*, 2010), are missing in I. There is almost no *intramolecular* C–H···O interaction in I, judged from the C5–C1–C6–O torsion angle of 41.5 (2)°, which deviates significantly from that reported for such a hydrogen-bonding system. In its 4,4'-isomer, the corresponding H3···O8 distance is 2.52 (1) Å and the C3–C4–C7–O8 torsion angle measures -21.9 (1)°.

Instead of *intermolecular* C–H···N and C–H···F interactions, stabilization of the structure of I is likely due to effective *intermolecular* C–H..π and F···F interactions. The π-π stacking is the driving force towards crystallization, with two adjacent bpy layers at a distance of 3.512 (2) Å. On top of this, the C6–H6A..π hydrogen bonding interaction has been observed between the methylene H atom and one of the adjacent bpy rings on the a-translation related direction. As shown in Table 1, the distance of H6A to bpy plane is 2.57 (2) Å, making less than 10° with the vector of H6A to centroid of the bpy ring. The terminal CF_3_ groups shown in Fig. 2 are then fixed in crystalline state by the F···F interaction between two adjacent stacking layers. The F1···F3' distance is 2.857 (2) Å, the C8–F1···F3' angle 101.4 (1)°, and the C8–F3···F1" angle 166.0 (1)°.

In particular, the weak C4–H4···H4'–C4' (Wolstenholme *et al.*, 2006) interaction shown in Fig. 2 has also been identified with H4···H4' distance of 2.32 (3) Å and the C4–H4···H4' angle of 113 (2)°. The C4–H4···H4'–C4' interaction connects two neighboring π stacking piles. Inside one π stacking pile, the π..π stacking distance between consecutive layers is 3.512 (2) Å, whereas the shifting step between two neighboring π stacking piles is 1.448 (2) Å. It is believed that this rare C–H···H–C supramolecular interaction seems to be derived from the dipole-induced interactions, defined by Wolstenholme, on the symmetry-related hydrogen atoms.

### Experimental

5,5'-bis(CF_3_CH_2_OCH_2_)-2,2'-bpy, (I), was prepared according to the general procedure described in Lu *et al.*, (2007). The crude product was further purified by vacuum sublimation or chromatography to obtain the title compound as a colorless solid. Full characterization data are listed below.

Analytical data of (I): Yield 76 %, m.p. = 393 K. ^1^H NMR (500 MHz, d-DMSO, room temperature), δ(ppm) Pyridine ring H: 8.66 s, H6, 2H), 8.39 (d, H4, ^3^J_HH_=8.24 Hz, 2H), 7.92 (d, H3, ^3^J_HH_=8.24 Hz, 2H), 4.77 (s, bpy-CH_2_, 4H), 4.17 (q, -OCH_2_CF_3_, ^3^J_HF_=9.34 Hz, 4H); ^19^F NMR (470.5 MHz, d-DMSO, room temperature), δ (ppm) -73.1 (t, -CH_2_CF_3_, ^3^J_HF_ = 9.7 Hz, 6F); ^13^C NMR (126 MHz,d-DMSO, room temperature) δ (ppm) 120.3, 133.1, 136.9, 148.7, 154.7 (s, bpy, 10C), 121.1-127.8 (q, -CF_3_, ^1^J_CF_ = 279.6 Hz, 2C), 70.6 (s, bpy-CH_2_, 2C), 66.8 (q, -CH_2_CF_3_, ^2^J_CF_ = 33.2 Hz, 2C).

GC/MS (M/e) : M^+^ = 380, (M-C_2_H_3_F_3_O)^+^ = 281, [M-(C_2_H_3_F_3_O)_2_]^+^= 182, (C_6_H_5_N)^+^ = 91.

FT-IR (cm^-1^): 1601.5, 1553.8, 1469.4, 1360.1 (bpy-ring, *m*), 1155.8, 1122.6 (CF_2_ stretch, *s*).

Recrystallization proceeded with dissolution of  I in DMSO to form a saturated solution, to which the water overlayer (5 cm^3^) was added. Solvent diffusion over a period of ten days at 298 K afforded needle shaped crystals.

### Refinement

The diffraction data were collected at 100K employing a Bruker CCD diffractometer; the structure was solved by successive Fourier maps. All H atoms were located at the end of anisotropic refinements and refined isotropically to convergence.

### Crystal data

**Table:**

C_16_H_14_F_6_N_2_O_2_	*Z* = 1
*M**_r_* = 380.29	*F*(000) = 194
Triclinic, *P*1	*D*_x_ = 1.605 Mg m^−^^3^*D*_m_ = 1.53 Mg m^−^^3^*D*_m_ measured by w/v
Hall symbol: -P 1	Mo *K*α radiation, λ = 0.71073 Å
*a* = 4.6573 (2) Å	Cell parameters from 3201 reflections
*b* = 5.6842 (3) Å	θ = 2.6–27.1°
*c* = 15.7273 (8) Å	µ = 0.15 mm^−^^1^
α = 94.298 (3)°	*T* = 100 K
β = 98.473 (3)°	Prism, colourless
γ = 105.689 (4)°	0.2 × 0.14 × 0.12 mm
*V* = 393.57 (3) Å^3^	

### Data collection

**Table:**

Bruker APEXII CCD area-detector diffractometer	1348 reflections with *I* > 2σ(*I*)
graphite	*R*_int_ = 0.024
φ and ω scans	θ_max_ = 26.4°, θ_min_ = 1.3°
Absorption correction: multi-scan (*SADABS*; Bruker, 2008)	*h* = −5→5
*T*_min_ = 0.664, *T*_max_ = 0.746	*k* = −7→7
6627 measured reflections	*l* = −19→19
1577 independent reflections	

### Refinement

**Table:**

Refinement on *F*^2^	Primary atom site location: structure-invariant direct methods
Least-squares matrix: full	Secondary atom site location: difference Fourier map
*R*[*F*^2^ > 2σ(*F*^2^)] = 0.031	Hydrogen site location: inferred from neighbouring sites
*wR*(*F*^2^) = 0.104	All H-atom parameters refined
*S* = 1.12	*w* = 1/[σ^2^(*F*_o_^2^) + (0.0595*P*)^2^ + 0.094*P*] where *P* = (*F*_o_^2^ + 2*F*_c_^2^)/3
1577 reflections	(Δ/σ)_max_ < 0.001
146 parameters	Δρ_max_ = 0.29 e Å^−^^3^
0 restraints	Δρ_min_ = −0.27 e Å^−^^3^

### Special details

**Table:**

Geometry. All esds (except the esd in the dihedral angle between two l.s. planes) are estimated using the full covariance matrix. The cell esds are taken into account individually in the estimation of esds in distances, angles and torsion angles; correlations between esds in cell parameters are only used when they are defined by crystal symmetry. An approximate (isotropic) treatment of cell esds is used for estimating esds involving l.s. planes.
Refinement. Refinement of F^2^ against ALL reflections. The weighted R-factor wR and goodness of fit S are based on F^2^, conventional R-factors R are based on F, with F set to zero for negative F^2^. The threshold expression of F^2^ > 2sigma(F^2^) is used only for calculating R-factors(gt) etc. and is not relevant to the choice of reflections for refinement. R-factors based on F^2^ are statistically about twice as large as those based on F, and R- factors based on ALL data will be even larger.

### Fractional atomic coordinates and isotropic or equivalent isotropic displacement parameters (Å^2^)

**Table:**

	*x*	*y*	*z*	*U*_iso_*/*U*_eq_	
F1	1.8989 (2)	1.22574 (16)	0.98212 (6)	0.0271 (3)	
F2	1.8270 (2)	1.35589 (16)	0.85765 (6)	0.0299 (3)	
F3	1.4537 (2)	1.23169 (17)	0.92410 (6)	0.0294 (3)	
O	1.4170 (2)	0.88799 (19)	0.79273 (7)	0.0225 (3)	
N	0.6609 (3)	0.3432 (2)	0.58189 (8)	0.0173 (3)	
C1	1.1096 (3)	0.6201 (3)	0.67039 (9)	0.0160 (3)	
C2	0.8935 (3)	0.3918 (3)	0.64727 (10)	0.0174 (3)	
C3	0.6289 (3)	0.5286 (2)	0.53652 (8)	0.0144 (3)	
C4	0.8268 (3)	0.7658 (3)	0.55708 (10)	0.0181 (3)	
C5	1.0718 (3)	0.8098 (3)	0.62300 (10)	0.0189 (3)	
C6	1.3693 (3)	0.6554 (3)	0.74291 (10)	0.0187 (3)	
C7	1.6509 (4)	0.9271 (3)	0.86434 (10)	0.0214 (4)	
C8	1.7066 (3)	1.1854 (3)	0.90685 (10)	0.0204 (3)	
H2	0.903 (4)	0.259 (4)	0.6804 (12)	0.025 (5)*	
H4	0.789 (4)	0.887 (3)	0.5248 (11)	0.023 (4)*	
H5	1.215 (4)	0.970 (4)	0.6343 (12)	0.029 (5)*	
H6A	1.556 (4)	0.658 (3)	0.7197 (11)	0.021 (4)*	
H6B	1.327 (4)	0.527 (3)	0.7796 (12)	0.024 (4)*	
H7A	1.594 (4)	0.813 (4)	0.9065 (12)	0.026 (5)*	
H7B	1.843 (4)	0.916 (3)	0.8461 (12)	0.028 (5)*	

### Atomic displacement parameters (Å^2^)

**Table:**

	*U*^11^	*U*^22^	*U*^33^	*U*^12^	*U*^13^	*U*^23^
F1	0.0318 (5)	0.0225 (5)	0.0219 (5)	0.0071 (4)	−0.0072 (4)	−0.0029 (4)
F2	0.0381 (6)	0.0217 (5)	0.0283 (5)	0.0050 (4)	0.0051 (4)	0.0072 (4)
F3	0.0266 (5)	0.0305 (5)	0.0318 (5)	0.0125 (4)	0.0036 (4)	−0.0048 (4)
O	0.0264 (6)	0.0187 (5)	0.0205 (6)	0.0106 (4)	−0.0067 (5)	−0.0044 (4)
N	0.0208 (6)	0.0119 (6)	0.0188 (6)	0.0044 (5)	0.0024 (5)	0.0025 (5)
C1	0.0161 (7)	0.0164 (7)	0.0168 (7)	0.0063 (6)	0.0046 (6)	−0.0002 (6)
C2	0.0223 (8)	0.0126 (7)	0.0182 (7)	0.0065 (6)	0.0031 (6)	0.0027 (5)
C3	0.0162 (7)	0.0125 (7)	0.0156 (7)	0.0044 (5)	0.0054 (6)	0.0023 (5)
C4	0.0208 (7)	0.0133 (7)	0.0198 (7)	0.0035 (6)	0.0026 (6)	0.0055 (6)
C5	0.0187 (7)	0.0139 (7)	0.0217 (8)	0.0006 (6)	0.0028 (6)	0.0022 (6)
C6	0.0192 (7)	0.0156 (7)	0.0210 (8)	0.0061 (6)	0.0015 (6)	−0.0002 (6)
C7	0.0236 (8)	0.0205 (8)	0.0188 (8)	0.0084 (6)	−0.0033 (7)	0.0004 (6)
C8	0.0212 (8)	0.0209 (8)	0.0183 (7)	0.0074 (6)	−0.0008 (6)	0.0015 (6)

### Geometric parameters (Å, °)

**Table:**

F1—C8	1.3386 (17)	C3—C4	1.396 (2)
F2—C8	1.3411 (18)	C3—C3^i^	1.481 (3)
F3—C8	1.3347 (18)	C4—C5	1.377 (2)
O—C7	1.4053 (18)	C4—H4	0.926 (19)
O—C6	1.4304 (17)	C5—H5	0.96 (2)
N—C2	1.3322 (19)	C6—H6A	0.990 (19)
N—C3	1.3446 (18)	C6—H6B	0.960 (19)
C1—C5	1.390 (2)	C7—C8	1.505 (2)
C1—C2	1.394 (2)	C7—H7A	0.98 (2)
C1—C6	1.494 (2)	C7—H7B	1.00 (2)
C2—H2	0.96 (2)		
			
C7—O—C6	111.24 (11)	O—C6—H6A	108.1 (10)
C2—N—C3	117.65 (12)	C1—C6—H6A	110.2 (10)
C5—C1—C2	117.07 (14)	O—C6—H6B	109.2 (11)
C5—C1—C6	122.24 (13)	C1—C6—H6B	110.7 (11)
C2—C1—C6	120.69 (13)	H6A—C6—H6B	109.9 (14)
N—C2—C1	124.41 (13)	O—C7—C8	107.27 (12)
N—C2—H2	115.6 (11)	O—C7—H7A	111.5 (11)
C1—C2—H2	119.9 (11)	C8—C7—H7A	108.4 (11)
N—C3—C4	122.04 (13)	O—C7—H7B	111.4 (10)
N—C3—C3^i^	117.10 (15)	C8—C7—H7B	107.4 (11)
C4—C3—C3^i^	120.85 (15)	H7A—C7—H7B	110.6 (16)
C5—C4—C3	119.25 (13)	F3—C8—F1	107.01 (12)
C5—C4—H4	122.6 (11)	F3—C8—F2	106.48 (12)
C3—C4—H4	118.2 (11)	F1—C8—F2	107.15 (12)
C4—C5—C1	119.49 (14)	F3—C8—C7	112.67 (13)
C4—C5—H5	119.0 (11)	F1—C8—C7	110.74 (12)
C1—C5—H5	121.5 (11)	F2—C8—C7	112.46 (13)
O—C6—C1	108.60 (11)		
			
C3—N—C2—C1	−1.6 (2)	C6—C1—C5—C4	−179.74 (13)
C5—C1—C2—N	1.9 (2)	C7—O—C6—C1	177.45 (12)
C6—C1—C2—N	−177.87 (13)	C5—C1—C6—O	41.49 (19)
C2—N—C3—C4	−1.1 (2)	C2—C1—C6—O	−138.72 (14)
C2—N—C3—C3^i^	179.43 (14)	C6—O—C7—C8	174.02 (13)
N—C3—C4—C5	3.4 (2)	O—C7—C8—F3	52.09 (17)
C3^i^—C3—C4—C5	−177.17 (15)	O—C7—C8—F1	171.89 (12)
C3—C4—C5—C1	−3.0 (2)	O—C7—C8—F2	−68.27 (17)
C2—C1—C5—C4	0.5 (2)		

Symmetry codes: (i) −*x*+1, −*y*+1, −*z*+1.

### Hydrogen-bond geometry (Å, °)

**Table:**

Cg is the centroid of the N,C1–C5 ring.

**Table:**

*D*—H···*A*	*D*—H	H···*A*	*D*···*A*	*D*—H···*A*
C4—H4···N^i^	0.925 (17)	2.464 (18)	2.809 (2)	102.3 (12)
C6—H6A···Cg^ii^	0.990 (19)	2.59	3.5089 (16)	155

Symmetry codes: (i) −*x*+1, −*y*+1, −*z*+1; (ii) *x*+1, *y*, *z*.

### Table 2 C—H···π contact for (I); hpd = H-atom-to-ring-plane distance, hcd = H-atom-to-ring-center distance, and sa = slippage angle (angle subtended by the hcd vector to the plane normal).

**Table:**

	hpd(Å)	hcd(Å)	sa(°)
C6-H6A···π^iii^	2.57 (2)	2.59 (2)	6.4 (2)

Symmetry code: (iii) x-1, y, z.
